# The Amazon continuum dataset: quantitative metagenomic and metatranscriptomic inventories of the Amazon River plume, June 2010

**DOI:** 10.1186/2049-2618-2-17

**Published:** 2014-05-15

**Authors:** Brandon M Satinsky, Brian L Zielinski, Mary Doherty, Christa B Smith, Shalabh Sharma, John H Paul, Byron C Crump, Mary Ann Moran

**Affiliations:** 1 Department of Microbiology, University of Georgia, Athens, GA 30602, USA; 2 College of Marine Science, University of South Florida, St. Petersburg, FL 33701, USA; 3 College of Earth, Ocean, and Atmospheric Science, Oregon State University, Corvallis, OR 97331, USA; 4 Department of Marine Sciences, University of Georgia, Athens, GA 30602, USA

**Keywords:** Amazon River plume, Metagenomics, Metatranscriptomics, Internal standard, Marine microbial communities

## Abstract

**Background:**

The Amazon River is by far the world’s largest in terms of volume and area, generating a fluvial export that accounts for about a fifth of riverine input into the world’s oceans. Marine microbial communities of the Western Tropical North Atlantic Ocean are strongly affected by the terrestrial materials carried by the Amazon plume, including dissolved (DOC) and particulate organic carbon (POC) and inorganic nutrients, with impacts on primary productivity and carbon sequestration.

**Results:**

We inventoried genes and transcripts at six stations in the Amazon River plume during June 2010. At each station, internal standard-spiked metagenomes, non-selective metatranscriptomes, and poly(A)-selective metatranscriptomes were obtained in duplicate for two discrete size fractions (0.2 to 2.0 μm and 2.0 to 156 μm) using 150 × 150 paired-end Illumina sequencing. Following quality control, the dataset contained 360 million reads of approximately 200 bp average size from Bacteria, Archaea, Eukarya, and viruses. Bacterial metagenomes and metatranscriptomes were dominated by *Synechococcus, Prochlorococcus*, SAR11, SAR116, and SAR86, with high contributions from SAR324 and Verrucomicrobia at some stations. Diatoms, green picophytoplankton, dinoflagellates, haptophytes, and copepods dominated the eukaryotic genes and transcripts. Gene expression ratios differed by station, size fraction, and microbial group, with transcription levels varying over three orders of magnitude across taxa and environments.

**Conclusions:**

This first comprehensive inventory of microbial genes and transcripts, benchmarked with internal standards for full quantitation, is generating novel insights into biogeochemical processes of the Amazon plume and improving prediction of climate change impacts on the marine biosphere.

## Background

The Amazon River runs nearly 6,500 km across the South American continent before emptying into the Western Tropical North Atlantic Ocean; in terms of both volume and watershed area it is the world’s largest riverine system [1]. The river carries a significant load of terrestrially-derived nutrients to the ocean, and this has global consequences on marine primary productivity and carbon sequestration [2,3]. Productive phytoplankton blooms harboring cyanobacteria, coastal diatom species, and oceanic diatoms with endosymbiotic diazotrophs take advantage of the riverine nutrient supplements and enhance carbon export from the upper ocean to deeper waters via sinking particles [3,4]. Heterotrophic bacteria also remineralize organic nutrients in the plume, further fueling primary production and increasing the flux of organic material to deep water.

**Figure 1:**
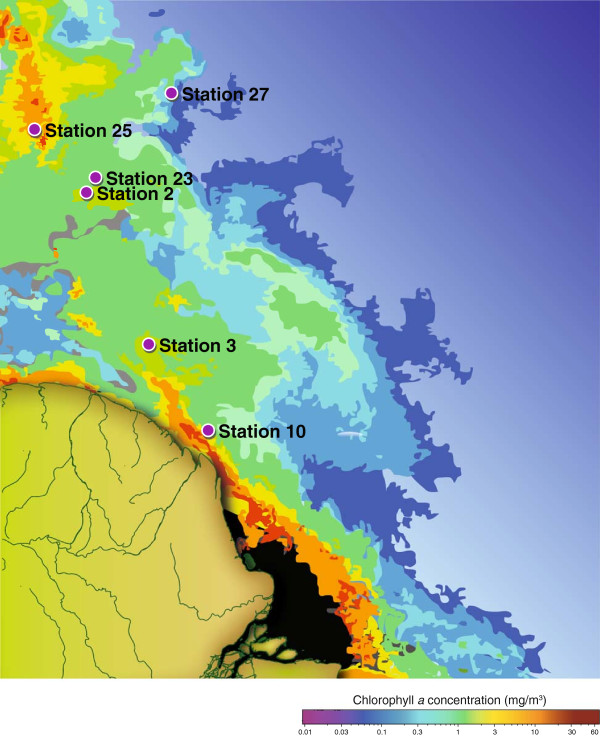
Location of sampling sites in the Amazon River plume in June, 2010.

We inventoried the microbial genes and transcripts at six stations in the Amazon River plume aboard the R/V *Knorr* between 22 May and 25 June, 2010 (Figure 1) using Illumina sequencing with 150 × 150 bp overlapping paired-end reads. Metagenomic and metatranscriptomic data have typically been analyzed within a relative framework (that is, % of metagenome and % of metatranscriptome), but this approach is problematic for dynamic communities because a change in the abundance of one type of gene or transcript imposes a change in the percent contribution of the others. By incorporating internal standards, we are able to assess meta-omics datasets within an absolute framework that facilitates comparisons of communities sampled at different times and places in the environment. In the Amazon plume sequence libraries, known copy numbers of internal standards were added at the initiation of sample processing and consisted of genomic DNA from an exotic bacterium for the metagenomes (*Thermus thermophilus* HB8) and artificial mRNAs and poly(A)-tailed mRNAs for the metatranscriptomes; these standards were identified, counted, and removed from the natural sequences during quality control steps.

For each station, metagenomes and non-selective metatranscriptomes were each obtained in duplicate for two discrete size fractions (0.2 to 2.0 μm and 2.0 to 156 μm), while poly(A)-selective metatranscriptomes were obtained in duplicate only for the 2.0 to 156 μm size fraction (to increase coverage of the eukaryotic community), resulting in a total of 60 datasets (6 stations x 5 data types × 2 replicates) (Table 1). The data collection consisted of 360 million reads following quality control (removal of poor quality reads, removal of rRNAs from metatranscriptomes, removal of internal standards, and joining of overlapping 150 bp paired ends) and provides an unprecedented view of the metabolic functions of the Bacteria, Archaea, and Eukarya mediating carbon and nutrient cycling in the Amazon River plume.

**Table 1:** **Number and types of libraries and reads obtained in the Amazon Continuum Project, June 2010, R/V *Knorr***

	**Metagenomes**	**Non-selective metatranscriptomes**	**Poly(A)-selective metatranscriptomes**
Data type	Total community DNA	Total community mRNA	Eukaryotic community mRNA^a^
# Stations sampled	6	6	6
# Size fractions sampled	2	2	1
# Replicates	2	2	2
# Samples	24	24	12
# Raw reads	3.68 × 10^8^	8.12 × 10^8^	4.61 × 10^8^
# Joined reads post QC	9.50 × 10^7^	1.62 × 10^8^	1.01 × 10^8^
Average joined read length (bp)	205	190	185
# rRNA reads	-	9.53 × 10^7^	2.34 × 10^5^
# Potential protein-encoding reads	9.44 × 10^7^	6.52 × 10^7^	9.86 × 10^7^

^a^The selective metatranscriptomes captured poly(A)**-**tailed transcripts and are therefore systematically biased against transcripts from eukaryotic organelles. #, number of; QC, quality control.

## Methods

Detailed sample collection and processing methodology can be found in Additional file 1. Sample sites in the Amazon River plume were chosen to represent a range of salinity, nutrient concentrations, and microbial communities (Additional file 2). Microbial cells were collected by filtration and preserved in RNAlater (Applied Biosystems, Austin, TX, USA). During sample processing, internal standards were added to each sample prior to cell lysis. Samples collected for non-selective metatranscriptomics were processed by extracting total RNA, removing residual DNA, depleting rRNA, linearly amplifying the remaining transcripts, and making double-stranded cDNA for library preparation and sequencing. Poly(A)-selective metatranscriptome samples were processed similarly except that poly(A)-tailed mRNAs were selectively isolated, eliminating the need for rRNA depletion steps. Metagenomic samples were processed by extracting DNA and removing residual proteins and RNA. Following sample processing, cDNA or DNA was sheared and libraries were constructed for paired-end sequencing (150 × 150) using either the Genome Analyzer IIx, HiSeq 2000, MiSeq, or HiSeq 2500 platform (Illumina Inc., San Diego, CA).

From 60 samples, we obtained 8.21 × 10^8^ raw sequences containing 1.23 × 10^11^ nt. Following sequence quality control, 3.59 × 10^8^ reads with a mean length of 195 bp were obtained. Internal standards were quantified and removed, along with any remaining rRNA sequences. Remaining reads were annotated against the RefSeq Protein database or a custom marine database using RAPSearch2 [5], and abundance per liter was calculated based on internal standard recovery [6] (Additional file 2).

Biological and chemical data measured concurrently with sample collection provides environmental context for sequence data. These metadata include temperature, salinity, oxygen concentration, irradiance, chlorophyll concentration, nutrient concentrations, and bacterial abundance and production (Additional file 2). Datasets describing the phytoplankton communities and other features of the June 2010 plume ecosystem have been previously published [1,4,7,8].

## Quality assurance

The She-ra program [9] was used to join the paired-end Illumina reads using the default parameters and a quality metric score of 0.5. Seqtrim [10] was used to trim the joined reads using the default parameters. rRNA and internal standard sequences were identified in the metatranscriptomes using a Blastn search against a custom database containing representative rRNA sequences and internal standard sequences; sequences with a bit score ≥ 50 were identified as either rRNA or internal standards and removed from the datasets. Internal standards were identified in metagenomes by first performing a Blastn search (bit score cutoff ≥ 50) against the *T. thermophilus* HB8 genome. Hits were subsequently queried against the RefSeq protein database using Blastx (bit score cutoff ≥ 40) to identify and quantify all *T. thermophilus* HB8 protein encoding reads, and these reads were removed from the datasets.

## Initial findings

Metagenomic reads from surface waters of the six Amazon River plume stations were assigned to bacterial, archaeal, eukaryotic, and viral taxa based on best hits to reference genomes. Among autotrophic bacteria, *Synechococcus* was the largest contributor to the metagenomes at locations closest to the river mouth (Stations 10, 3; approximately 1.5 × 10^12^ genes L^−1^) and was replaced by *Prochlorococcus* at more oceanic locations (Stations 25, 27) (Table 2). Among heterotrophic bacteria, SAR86 had the largest gene abundance closest to the river mouth (Station 10; approximately 8.6 × 10^11^ genes L^−1^). SAR11 clade members (HTCC7211, HIMB5) were also abundant here, and became the dominant contributor of heterotrophic bacterial genes at more oceanic stations (up to 5.7 × 10^12^ genes L^−1^) (Table 2). Genes binning to SAR324 genomes were abundant at three stations (Station 2, 3, and 23; Table 2), with the Amazon plume sequences aligning with heterotrophic members of this group [11]. Station 2 had a distinctive bacterial community relative to the other plume stations, dominated by genes from Verrucomicrobia related to *Coraliomargarita akajimensis* DSM 45221 and strain DG1235 and with substantial contributions from SAR116 taxa (IMCC1322, HIMB100). *Coraliomargarita akajimensis* DSM 45221 was also among the most abundant genome bins at Station 25 (Table 2).

**Table 2:** Reference genome bins garnering the most metagenomic reads, organized by station and domain (top 10 Bacteria, 4 Eukarya, 2 Archaea, and 2 viruses)

**Domain**	**Taxon**	**Genes L**^**−1**^	**Domain**	**Taxon**	**Genes L**^**−1**^
Station 10					
Bacteria	*Synechococcus* sp. CB0205	1.46 × 10^12^	Eukarya	*Thalassiosira oceanica* CCMP1005	1.26 × 10^11^
Bacteria	SAR86 E	4.65 × 10^11^	Eukarya	*Micromonas* sp. RCC299	8.27 × 10^10^
Bacteria	SAR86 D	2.55 × 10^11^	Eukarya	*Tetrahymena thermophila* SB210	2.41 × 10^10^
Bacteria	Alphaproteobacterium HIMB5	2.32 × 10^11^	Eukarya	*Strombidinopsis* sp. SopsisLIS2011	1.67 × 10^10^
Bacteria	*Cand*. Pelagibacter sp. HTCC7211	2.22 × 10^11^			
Bacteria	*Cand*. Pelagibacter ubique	1.54 × 10^11^	Archaea	*Nitrosopumilus maritimus* SCM1	1.73 × 10^10^
Bacteria	SAR86 C	1.39 × 10^11^	Archaea	*Cand*. Nitrosopumilus koreensis AR1	1.02 × 10^10^
Bacteria	Gammaproteobacterium HIMB55	1.33 × 10^11^			
Bacteria	*Synechococcus* sp. CB0101	1.19 × 10^11^	Virus	*Synechococcus* phage S-RSM4	1.74 × 10^11^
Bacteria	Gammaproteobacterium HIMB30	1.15 × 10^11^	Virus	*Synechococcus* phage S-SKS1	1.74 × 10^11^
Station 3					
Bacteria	*Cand*. Pelagibacter sp. HTCC7211	2.79 × 10^11^	Eukarya	*Micromonas* sp. RCC299	2.28 × 10^10^
Bacteria	Alphaproteobacterium HIMB5	2.02 × 10^11^	Eukarya	*Tetrahymena thermophila* SB210	4.93 × 10^9^
Bacteria	SAR86 D	1.64 × 10^11^	Eukarya	*Alexandrium tamarense* CCMP1771	3.71 × 10^9^
Bacteria	SAR86 E	1.33 × 10^11^	Eukarya	*Thalassiosira oceanica* CCMP1005	3.49 × 10^9^
Bacteria	*Cand*. Pelagibacter ubique	1.17 × 10^11^			
Bacteria	Alphaproteobacterium HIMB59	9.29 × 10^10^	Archaea	*Nitrosopumilus maritimus* SCM1	3.01 × 10^9^
Bacteria	SAR86 C	8.58 × 10^10^	Archaea	*Cand*. Nitrosoarchaeum limnia	2.31 × 10^9^
Bacteria	*Synechococcus* sp. WH 8109	7.33 × 10^10^			
Bacteria	*Cand*. Pelagibacter ubique HTCC1062	6.37 × 10^10^	Virus	*Synechococcus* phage S-RSM4	6.70 × 10^10^
Bacteria	SAR324 JCVI-SC AAA005	5.06 × 10^10^	Virus	*Synechococcus* phage S-SKS1	2.57 × 10^10^
Station 2					
Bacteria	*Coraliomargarita akajimensis* DSM 45221	3.31 × 10^12^	Eukarya	*Phaeocystis antarctica*	1.51 × 10^12^
Bacteria	*Cand*. Puniceispirillum marinum IMCC1322	7.46 × 10^11^	Eukarya	*Phytophthora sojae*	1.02 × 10^12^
Bacteria	Gammaproteobacterium HIMB55	6.13 × 10^11^	Eukarya	*Emiliania hu×leyi*	9.44 × 10^11^
Bacteria	*Synechococcus* sp. WH 8109	6.07 × 10^11^	Eukarya	*Aplanochytrium kerguelense*	7.60 × 10^11^
Bacteria	SAR116 HIMB100	5.95 × 10^11^			
Bacteria	*Cand*. Pelagibacter sp. HTCC7211	5.09 × 10^11^	Archaea	*Cand*. Nitrosopumilus salaria	1.54 × 10^10^
Bacteria	SAR324 JCVI-SC AAA005	4.61 × 10^11^	Archaea	*Methanomassiliicoccus* sp. M × 1-Issoire	5.89 × 10^9^
Bacteria	Gammaproteobacterium HTCC2207	3.91 × 10^11^			
Bacteria	Verrucomicrobiae DG1235	3.39 × 10^11^	Virus	*Synechococcus* phage S-RIP1	9.55 × 10^8^
Bacteria	*Prochlorococcus marinus* str. AS9601	3.16 × 10^11^	Virus	*Phaeocystis globosa* virus	6.20 × 10^8^
Station 23					
Bacteria	*Cand*. Pelagibacter sp. HTCC7211	1.36 × 10^12^	Eukarya	*Tetrahymena thermophila* SB210	2.96 × 10^10^
Bacteria	Alphaproteobacterium HIMB5	9.43 × 10^11^	Eukarya	*Protocruzia adherens* Boccale	2.84 × 10^10^
Bacteria	SAR86 D	9.31 × 10^11^	Eukarya	*Strombidinopsis* sp. SopsisLIS2011	2.82 × 10^10^
Bacteria	Alphaproteobacterium HIMB59	7.03 × 10^11^	Eukarya	*Pseudo-nitzschia multiseries*	1.79 × 10^10^
Bacteria	SAR86 E	6.95 × 10^11^			
Bacteria	*Cand*. Pelagibacter ubique	5.17 × 10^11^	Archaea	*Methanosarcina acetivorans* C2A	1.60 × 10^9^
Bacteria	SAR86 C	4.69 × 10^11^	Archaea	*Methanosarcina barkeri* str. Fusaro	1.37 × 10^9^
Bacteria	*Cand*. Pelagibacter ubique HTCC1062	2.74 × 10^11^			
Bacteria	SAR324 JCVI-SC AAA005	2.33 × 10^11^	Virus	*Phaeocystis globosa* virus	1.01 × 10^11^
Bacteria	Alphaproteobacterium HIMB114	2.31 × 10^11^	Virus	*Synechococcus* phage S-SM2	4.62 × 10^10^
Station 25					
Bacteria	*Cand*. Pelagibacter sp. HTCC7211	6.83 × 10^11^	Eukarya	*Pyraminomonas obovata* CCMP722	8.58 × 10^9^
Bacteria	Alphaproteobacterium HIMB5	4.13 × 10^11^	Eukarya	*Phaeocystis antarctica*	6.34 × 10^9^
Bacteria	Alphaproteobacterium HIMB59	2.35 × 10^11^	Eukarya	*Thalassiosira oceanica* CCMP1005	5.67 × 10^9^
Bacteria	*Cand*. Pelagibacter ubique	2.07 × 10^11^	Eukarya	*Volvox carteri* f. nagariensis	4.93 × 10^9^
Bacteria	*Prochlorococcus marinus* str. AS9601	1.87 × 10^11^			
Bacteria	*Prochlorococcus marinus* str. MIT 9301	1.70 × 10^11^	Archaea	*Methanosarcina acetivorans* C2A	9.95 × 10^8^
Bacteria	SAR86 E	1.67 × 10^11^	Archaea	*Methanomassiliicoccus* sp. M × 1-Issoire	8.48 × 10^8^
Bacteria	SAR86 D	1.61 × 10^11^			
Bacteria	*Coraliomargarita akajimensis* DSM 45221	1.45 × 10^11^	Virus	*Phaeocystis globosa* virus	4.57 × 10^10^
Bacteria	Gammaproteobacterium HTCC2207	1.29 × 10^11^	Virus	*Synechococcus* phage S-SM2	2.36 × 10^10^
Station 27					
Bacteria	*Prochlorococcus marinus* str. AS9601	9.43 × 10^12^	Eukarya	*Phaeocystis antarctica*	3.04 × 10^10^
Bacteria	*Prochlorococcus marinus* str. MIT 9301	8.49 × 10^12^	Eukarya	*Tetrahymena thermophila* SB210	2.25 × 10^10^
Bacteria	*Cand*. Pelagibacter sp. HTCC7211	5.70 × 10^12^	Eukarya	*Ale×andrium tamarense* CCMP1771	1.56 × 10^10^
Bacteria	*Prochlorococcus marinus* str. MIT 9215	4.46 × 10^12^	Eukarya	*Monosiga brevicollis*	1.35 × 10^10^
Bacteria	Alphaproteobacterium HIMB5	3.96 × 10^12^			
Bacteria	*Prochlorococcus marinus* str. MIT 9312	3.13 × 10^12^	Archaea	*Methanomassiliicoccus* sp. M×1-Issoire	8.73 × 10^9^
Bacteria	*Cand*. Pelagibacter ubique	2.22 × 10^12^	Archaea	*Aciduliprofundum* sp. MAR08-339	6.10 × 10^9^
Bacteria	*Prochlorococcus marinus*	1.75 × 10^12^			
Bacteria	*Cand*. Pelagibacter ubique HTCC1062	1.31 × 10^12^	Virus	*Prochlorococcus* phage P-SSM2	6.08 × 10^11^
Bacteria	Alphaproteobacterium HIMB59	1.21 × 10^12^	Virus	*Synechococcus* phage S-SM2	3.20 × 10^11^

Bacterial, archaeal, and viral reads were annotated against the NCBI RefSeq database. Eukaryotic reads were annotated against a custom database containing marine eukaryotic genomes and transcriptomes from NCBI and 112 of the Marine Microbial Eukaryote Transcriptome Sequencing Project datasets that were public at the time of analysis (http://marinemicroeukaryotes.org).

Among eukaryotic taxa, diatoms and the green alga *Micromonas* contributed the greatest number of genes at lower salinities, while Haptophytes (binning to *Phaeocystis antarctica*), dinoflagellates (binning to *Alexandrium tamarense* CCMP1771) and relatives of the green alga *Pyraminomonas obovata* CCMP722 increased in importance at more saline stations (Table 2). Among Archaea, members of the ammonia-oxidizing genus *Nitrosopumilus* and related genera contributed the most genes at stations closest to the river mouth, although they were 100-fold lower in numbers compared to the most abundant bacterial taxa. There were very few archaeal genes at the outermost stations (Stations 25 and 27), and these binned largely to methanogen sequences. The viral sequences were dominated by cyanobacterial phages (Table 2).

**Figure 2:**
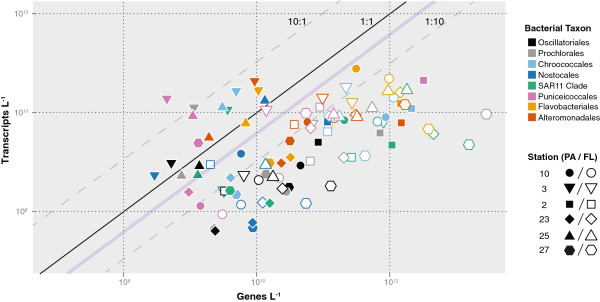
**Inventories of genes and transcripts for eight bacterial taxa in surface waters of the Amazon plume.** Symbols represent the mean of duplicate analyses at six stations, color-coded by taxon and size fraction (particle-associated or free-living). Lines indicate a 1:1 ratio of transcripts:genes (black) or 10:1 and 1:10 ratios (gray). The purple line indicates the ratio of transcripts:genes for exponentially growing laboratory cultures of *Escherichia coli*[12,13]. Dominant bacterial groups are as follows: Oscillatoriales = *Trichodesmium*; Prochlorales = *Prochlorococcus*; Chroococcales = *Synechococcus*; Nostocales = *Richelia*; Puniceicoccales = Verrucomicrobia.

Patterns of gene and transcript abundance provided insights into transcriptional activity by taxon and habitat (that is, cells that were free-living versus those that were particle-associated) for the dominant bacterial groups. Particle-associated Verrucomicrobia (Order Puniceicoccales) maintained cellular transcript inventories of up to 14 transcripts/gene for particle-associated cells and averaged 2 transcripts/gene overall (Figure 2). In contrast, members of the Flavobacteria class averaged < 0.5 transcripts/gene. Particle-associated cells in each of these major taxa typically had more transcripts per gene copy than did free-living cells (averaging 2.0 versus 0.15 transcripts/gene) (Figure 2). Abundance of transcripts originating from particle-associated versus free-living bacteria varied along the plume, with mRNAs from free-living cells contributing only 30 to 60% of the metatranscriptome in landward stations, but > 90% at outer plume stations. Environmental data (Additional file 2) indicate that Station 10 had the lowest salinity (22.6) and Station 27 the highest (36.0). Station 10 was the most strongly influenced by riverine inputs, particularly of inorganic nitrogen.

## Future directions

The Amazon River plume is immense in scale and sensitive to anthropogenic forcing. This multi-omics dataset is the first of four high-throughput metagenomic and metatranscriptomic sequence collections being produced for the Amazon River Continuum as part of the ANACONDAS and ROCA projects (http://amazoncontinuum.org). These projects aim to improve predictive capabilities for climate change impacts on the marine biosphere, focusing on the Amazon ecosystem, and to better our understanding of feedbacks on the carbon cycle. Processes in the river and ocean are tightly linked from physical, biological, and biogeochemical perspectives. Thus, the complete data collection will include two datasets from the Amazon plume (June 2010 and July 2013) and two from the Amazon River (Óbidos to Macapá and Belém; June 2011 and July 2013). These high-coverage, size-discrete, and replicated datasets are all benchmarked with internal genomic and mRNA standards for comparative quantitative metagenomics and metatranscriptomics. Insights from these meta-omics datasets are enhancing predictive capabilities regarding the interplay between marine microbial communities, biogeochemical cycling, and carbon sequestration in the ocean.

## Availability of supporting data

Sequences from June 2012 Amazon Continuum study are available from NCBI under accession numbers [SRP039390] (metagenomes), [SRP037995] (non-selective metatranscriptomes), and [SRP039544] (poly(A)-selected metatranscriptomes). The NCBI sequences are fastq files from which internal standard sequences and rRNA sequences (metatranscriptomes only) have been removed prior to deposition. Sequences are also available at the Community Cyberinfrastructure for Advanced Microbial Ecology Research and Analysis (CAMERA) database under project number CAM_P_0001194. The CAMERA sequences are QC’d fasta files of joined paired-end reads, also with internal standards and rRNA sequences (metatranscriptomes only) removed. Metadata accompanying the omics datasets are provided in Additional file 2. ANACONDAS and ROCA project data are also available at the BCO-DMO data repository (http://www.bco-dmo.org/project/2097).

## Abbreviations

bp: base pairs; DOC: dissolved organic carbon; nt: nucleotides; POC: particulate organic carbon.

## Competing interests

The authors declare that they have no competing interests.

## Authors’ contributions

BMS: conception and design of protocols, sample processing, data analysis, writing and final approval of the manuscript. BLZ: sample collection, sample processing, critical revision and final approval of the manuscript. MD: sample processing, protocol design, critical revision and final approval of the manuscript. CBS: sample processing, protocol design, critical revision and final approval of the manuscript. SS: data analysis, critical revision and final approval of the manuscript. JHP: critical revision and final approval of the manuscript. BCC: design of protocols, data analysis, critical revision and final approval of the manuscript. MAM: conception and design of protocols, data analysis, writing and final approval of the manuscript. All authors read and approved the final manuscript.

## Supplementary Material

Additional file 1 **Detailed methods.** Description of metagenome and metatranscriptome sample processing, sequencing, and data analysis, including internal standard additions and analysis. Click here for file

Additional file 2 **Metadata.** Metadata accompanying the metagenomic and metatranscriptomic datasets, including sample station locations,environmental conditions and library sizes and statistics. Click here for file

## References

[B1] Coles VJ Brooks MT Hopkins J Stukel MR Yager PL Hood RR The pathways and properties of the Amazon River plume in the tropical north Atlantic ocean J Geophys Res: Oceans 2013 118 6894 6913 10.1002/2013JC008981

[B2] Richey JE Nobre C Deser C Amazon River discharge and climate variability: 1903 to 1985 Science 1989 246 101 103 10.1126/science.246.4926.101 17837767

[B3] Subramaniam A Yager PL Carpenter EJ Mahaffey C Bjorkman K Cooley S Kustka AB Montoya JP Sanudo-Wilhelmy SA Shipe R Capone DG Amazon River enhances diazotrophy and carbon sequestration in the tropical north Atlantic ocean Proc Natl Acad Sci U S A 2008 105 10460 10465 10.1073/pnas.0710279105 18647838 PMC2480616

[B4] Goes JI Gomes HR Chekalyuk AM Carpenter EJ Montoya JP Coles VJ Yager PL Berelson WM Capone DG Foster RA Steinberg DK Subramaniam A Hafez MA Influence of the Amazon River discharge on the biogeography of phytoplankton communities in the Western Tropical North Atlantic Prog Oceanogr 2014 120 29 40

[B5] Zhao Y Tang H Ye Y RAPSearch2: a fast and memory-efficient protein similarity search tool for next-generation sequencing data Bioinformatics 2012 28 125 126 10.1093/bioinformatics/btr595 22039206 PMC3244761

[B6] Satinsky BM Gifford SM Crump BC Moran MA Edward FD Use of internal standards for quantitative metatranscriptome and metagenome analysis Methods in Enzymology 2013 531 12 Burlington, MA: Academic 237 250 24060124 10.1016/B978-0-12-407863-5.00012-5

[B7] Barada LP Cutter L Montoya JP Webb EA Capone DG Sanudo-Wilhelmy SA The distribution of thiamin and pyridoxine in the Western Tropical North Atlantic Amazon river plume Front Microbiol 2013 4 25 23471170 10.3389/fmicb.2013.00025 PMC3590742

[B8] Chong LS Berelson WM McManus J Hammond DE Rollins NE Yager PL Carbon and biogenic silica export influenced by the Amazon River Plume: patterns of remineralization in deep-sea sediments Deep Sea Res Part I: Oceanogr Res Papers 2014 85 124 137

[B9] Rodrigue S Materna AC Timberlake SC Blackburn MC Malmstrom RR Alm EJ Chisholm SW Unlocking short read sequencing for metagenomics PLoS One 2010 5 e11840 10.1371/journal.pone.0011840 20676378 PMC2911387

[B10] Falgueras J Lara AJ Fernandez-Pozo N Canton FR Perez-Trabado G Claros MG SeqTrim: a high-throughput pipeline for pre-processing any type of sequence read BMC Bioinformatics 2010 11 38 10.1186/1471-2105-11-38 20089148 PMC2832897

[B11] Chitsaz H Yee-Greenbaum JL Tesler G Lombardo MJ Dupont CL Badger JH Novotny M Rusch DB Fraser LJ Gormley NA Schulz-Trieglaff O Smith GP Evers DJ Pevzner PA Lasken RS Efficient *de novo* assembly of single-cell bacterial genomes from short-read data sets Nat Biotechnol 2011 29 915 921 10.1038/nbt.1966 21926975 PMC3558281

[B12] Neidhardt FC Umbarger HE Neidhardt FC, Curtiss RIII, Ingraham JL, Lin ECC, Low KB, Magasanik B, Reznikoff WS, Riley M, Schaechter M, Umbarger HE Chemical composition of *Escherichia coli* Escherichia Coli and Salmonella Typhimurium: Cellular and Molecular Biology 1996 2 Washington, DC: ASM Press 13 16

[B13] Taniguchi Y Choi PJ Li GW Chen H Babu M Hearn J Emili A Xie XS Quantifying E. coli proteome and transcriptome with single-molecule sensitivity in single cells Science 2010 329 533 538 10.1126/science.1188308 20671182 PMC2922915

