# Supplementary material for: The Amazon continuum dataset: quantitative metagenomic and metatranscriptomic inventories of the Amazon River plume, June 2010
Source: Microbiome. 2014 May 15;2:17. doi: 10.1186/2049-2618-2-17 (PMC4039049; doi:10.1186/2049-2618-2-17)
Supplement: Additional file 1 — Detailed methods. Description of metagenome and metatranscriptome sample processing, sequencing, and data analysis, including internal standard additions and analysis. [file 2049-2618-2-17-S1.pdf]

The Amazon Continuum Dataset: Quantitative Metagenomic and Metatranscriptomic Inventories of the Amazon River Plume, June 2010

Brandon M. Satinsky, Brian L. Zielinski, Mary Doherty, Christa B. Smith, Shalabh Sharma, John H. Paul, Byron C. Crump, and Mary Ann Moran

Detailed Methods

Sample Collection

At each of the six stations selected for analysis, surface water was collected by gentle impeller pumping (modified Rule 1800 submersible sump pump) through 10 m of tygon tubing (3 cm) to the ship's deck where the water then flowed through a 156 μm mesh prefilter and was collected in 20 L carboys. The water was sequentially filtered (using a Masterflex peristaltic pump) through a 2.0 μm pore-size, 142 mm diameter polycarbonate (PCTE) membrane filter (Sterlitech Corporation, Kent, CWA) and a 0.22 μm pore-size, 142 mm diameter Supor membrane filter (Pall, Port Washington, NY). Duplicate samples were collected for each membrane size/type for total community metagenomics and metatranscriptomics. Duplicate 2.0 μm pore-size, 142 mm diameter polycarbonate (PCTE) membrane filters were collected for poly(A)-selective metatranscriptomics. After filtration, membranes were immediately submerged in RNAlater (Applied Biosystems, Austin, TX) in sterile 50 ml conical tubes, incubated at room temperature overnight, and then stored at -80°C until extraction. All filtration and stabilization was completed within 30 min of water collection, and the volume of filtrate passed through each membrane was recorded.

RNA Processing for Total Community Metatranscriptomes

Prior to RNA extraction, the filters were thawed, removed from the preservative solution, placed in Whirl-Pak® bags (Nasco, Fort Artkinson, WI), and flash-frozen in liquid nitrogen. RNA extraction and DNA removal were carried out as previously described [1-3]. For 0.2 – 2.0 μm samples, a lysis tube was prepared for each sample consisting of a sterile 50 ml conical tube containing 8 ml of RLT Lysis Solution (Qiagen, Valencia, CA), 3 g RNA PowerSoil beads (Mo-Bio, Carlsbad, CA), and internal standards (described below). For ≥ 2.0 μm samples, a lysis tube was prepared for each sample consisting of a sterile 50 ml conical tube containing 10 ml of RLT Lysis Solution, 1.5 ml of 100 μm zirconium beads (OPS Diagnostics, Lebanon, NJ, USA), and internal standards. Filters inside the bags were broken into small pieces using a rubber mallet and transferred to the lysis tubes. Tubes were vortexed for 10 min to lyse cells, and RNA was purified from cell lysate using an RNeasy Kit (Qiagen, Valencia, CA) followed by two successive treatments with the Turbo DNA-free kit (Invitrogen, Carlsbad, CA) to remove residual DNA. Ribosomal RNA (rRNA) was selectively removed using community-specific biotinylated-rRNA probes prepared from DNA collected simultaneously [4]. To maximize the removal of rRNA, probes were created for Bacterial and Archaeal 16S and 23S rRNA and Eukaryotic 18S and 28S rRNA. Probe-bound rRNA was removed via hybridization to streptavidin-coated magnetic beads (New England Biolabs, Ipswich, MA), and successful removal of rRNA from the samples was confirmed using either an Experion automated electrophoresis system (Bio-Rad Laboratories, Hercules, CA) or a Bioanalyzer (Agilent Technologies, Santa Clara, CA). rRNA-depleted samples were linearly amplified using the MessageAmp II-Bacteria Kit (Applied Biosystems, Austin, TX), and amplified mRNA was converted into cDNA using the Superscript III First Strand synthesis system (Invitrogen, Carlsbad, CA) with random primers, followed by the NEBnext mRNA second strand synthesis module (New England Biolabs, Ipswich, MA), both according to manufacturer protocols. Synthesized cDNA was purified using the QIAquick PCR purification kit (Qiagen, Valencia, CA)

followed by EtOH precipitation, resuspension in 100 µL of TE buffer, and storage at -80° C until library preparation for sequencing.

RNA Processing for Poly(A)-tail Selected Metatranscriptomes

To ensure sufficient coverage of eukaryotic transcriptomes, a second metatranscriptome protocol was used that selectively sequenced messages with poly(A) tails; this was carried out for the >2.0 um pore-size filter only. Samples were prepared as described above with the following exceptions. An internal poly(A)-tailed mRNA standard was added to each lysis tube (see below). Following lysis, poly(A)-tailed mRNA was isolated from total RNA using an Oligotex mRNA kit (Qiagen, Valencia, CA), and mRNA was linearly amplified with a MessageAmp II-aRNA Amplification Kit (Applied Biosystems, Austin, TX). Double stranded cDNA was prepared as described above except cDNA was purified using the DNA Clean and Concentrator -25 Kit (Zymo, Irvine, CA) with five volumes of DNA binding buffer.

DNA Processing for Metagenomes

DNA was extracted and purified as previously described [5-7] with some modification. Briefly, each filter was thawed, removed from the preservative solution, and rinsed three times in autoclaved, filter-sterilized, 0.1% phosphate-buffered saline (PBS) to remove any residual RNAlater. Each filter was shattered as described above and placed in a tube containing DNA extraction buffer [DEB: 0.1 M Tris-HCl (pH 8), 0.1 M Na-EDTA (pH 8), 0.1 M Na2H2PO4 (pH 8), 1.5 M NaCl, 5% CTAB]. All liquid from the rinses as well as the original RNAlater was pushed through a Sterivex-GP filter capsule (EMD Millipore, Billerica, MA), which was subsequently rinsed 3 times to salvage any lost cells. The capsule was opened and the filter sliced into pieces and added to the tube with the original membrane filter and an internal genomic DNA standard (described below). Following treatments with proteinase-K, lysozyme, and sodium dodecyl sulfate, DNA was purified via phenol:chloroform extraction and isopropanol precipitation.

Internal Standards

Omics processing included the addition of internal standards to allow for calculation of volume-based absolute copy numbers for each gene or transcript type, rather than just relative quantification (i.e., counts L^{-1} in addition to % of library) [1, 8]. Two mRNA standards without poly(A) tails (to mimic prokaryotic and organelle mRNAs) were synthesized by in vitro transcription using a method modified from [1]. The standards were constructed by linearizing a pTXB1 vector (New England Biolabs, Ipswich, MA) with NcoI restriction enzyme (New England Biolabs, Ipswich, MA) or pFN18A Halotag T7 Flexi Vector (Promega, Madison, WI) with BamHI restriction enzyme (New England Biolabs, Ipswich, MA). Each was purified by phenol:chloroform:isoamyl alcohol extraction and ethanol precipitation. The 5' nucleotide overhangs were removed using Mung Bean Nuclease (New England Biolabs, Ipswich, MA), followed by purification via phenol:chloroform:isoamyl alcohol extraction and ethanol precipitation. Complete digestion of the vector was confirmed on a 1% agarose gel. The DNA fragment was then transcribed in vitro using the Riboprobe in vitro Transcription System (Promega, Madison, WI) according to the manufacturer's protocol using a T7 RNA polymerase to create 916 nt (pTXB1 standard) or 970 nt (pFN18A) artificial transcripts. Residual DNA was removed using RQ1 RNase-Free DNase and the RNA was purified by phenol:chloroform:isoamyl alcohol extraction and ethanol precipitation. The RNA standards were quantified using the Quant-iT Ribogreen RNA Reagent and Kit (Invitrogen, Carlsbad, CA), and RNA nucleotide length was confirmed with an Experion automated electrophoresis system (Bio-Rad Laboratories, Hercules, CA). A known number of each standard (pTXB1 = 2.104×10^{10} copies; pFN18A = 1.172×10^{10} copies) was added independently to each lysis tube immediately prior to the addition of the sample filter.

An mRNA standard with a poly(A) tail (to mimic eukaryotic nuclear mRNA) was created from an HAP-1 Protolomerase viral gene. To create the standard, a 544 bp amplicon containing a poly(A) tail and a T7 promoter was produced from the template DNA through PCR. The PCR amplicons were then used as the template DNA for an in vitro transcription reaction to produce the resulting 499 nucleotide poly(A)-tailed mRNA. A known number of each standard (2.0×10^9 copies) was added to each tube immediately prior to lysis.

The genomic internal standard consisted of *Thermus thermophilus* DSM7039 [HB8] genomic DNA (American Type Culture Collection, Manassas, VA) added immediately prior to cell lysis. The amount of DNA standard added was estimated to be ~ 1% (8.4 ng per liter filtered) of sample DNA.

Sequencing

cDNA and DNA was sheared ultrasonically to ~200-250 bp fragments and TruSeq libraries (Illumina Inc., San Diego, CA) were constructed for paired-end (150 x 150) sequencing using the Illumina Genome Analyzer IIx, HiSeq2000, MiSeq, or HiSeq2500 platforms (Illumina Inc., San Diego, CA).

References

1. Gifford SM, Sharma S, Rinta-Kanto JM, Moran MA: **Quantitative analysis of a deeply sequenced marine microbial metatranscriptome.** *ISME J* 2011, **5:**461-472.
2. Poretsky RS, Gifford S, Rinta-Kanto J, Vila-Costa M, Moran MA: **Analyzing gene expression from marine microbial communities using environmental transcriptomics.** *J Vis Exp* 2009.
3. Poretsky RS, Hewson I, Sun S, Allen AE, Zehr JP, Moran MA: **Comparative day/night metatranscriptomic analysis of microbial communities in the North Pacific subtropical gyre.** *Environ Microbiol* 2009, **11:**1358-1375.
4. Stewart FJ, Ottesen EA, DeLong EF: **Development and quantitative analyses of a universal rRNA-subtraction protocol for microbial metatranscriptomics.** *ISME J* 2010, **4:**896-907.
5. Crump BC, Armbrust EV, Baross JA: **Phylogenetic analysis of particle-attached and free-living bacterial communities in the Columbia river, its estuary, and the adjacent coastal ocean.** *Appl Environ Microbiol* 1999, **65:**3192-3204.
6. Crump BC, Kling GW, Bahr M, Hobbie JE: **Bacterioplankton community shifts in an arctic lake correlate with seasonal changes in organic matter source.** *Appl Environ Microbiol* 2003, **69:**2253-2268.
7. Zhou J, Bruns MA, Tiedje JM: **DNA recovery from soils of diverse composition.** *Appl Environ Microbiol* 1996, **62:**316-322.
8. Satinsky BM, Gifford SM, Crump BC, Moran MA: **Chapter Twelve - Use of Internal Standards for Quantitative Metatranscriptome and Metagenome Analysis.** In *Methods in Enzymology. Volume* 531. Edited by Edward FD: Academic Press; 2013: 237-250
